# Gender difference in NASH susceptibility: Roles of hepatocyte *Ikkβ* and *Sult1e1*

**DOI:** 10.1371/journal.pone.0181052

**Published:** 2017-08-10

**Authors:** Noriko Matsushita, Mohamed T. Hassanein, Marcos Martinez-Clemente, Raul Lazaro, Samuel W. French, Wen Xie, Keane Lai, Michael Karin, Hidekazu Tsukamoto

**Affiliations:** 1 Southern California Research Center for ALPD and Cirrhosis of the University of Southern California, Los Angeles, California, United States of America; 2 Department of Pathology, Keck School of Medicine of the University of Southern California, Los Angeles, California, United States of America; 3 Harbor-UCLA Medical Center, Torrance, California, United States of America; 4 Center for Pharmacogenetics, Department of Pharmaceutical Sciences, University of Pittsburgh School of Pharmacy, Pittsburgh, Pennsylvania, United States of America; 5 Departments of Pharmacology and Pathology, University of California San Diego, La Jolla, California, United States of America; 6 Department of Veterans Affairs Greater Los Angeles Healthcare System, Los Angeles, California, United States of America; Bambino Gesù Children's Hospital, ITALY

## Abstract

Myeloid cell and hepatocyte IKKβ may mediate the genesis of obesity and insulin resistance in mice fed high fat diet. However, their gender-specific roles in the pathogenesis of non-alcoholic steatohepatitis (NASH) are not known. Here we demonstrate myeloid IKKβ deficiency prevents Western diet-induced obesity and visceral adiposity in females but not in males, and attenuates hyperglycemia, global IR, and NASH in both genders. In contrast, all metabolic sequela including NASH are aggravated by hepatocyte IKKβ deficiency (*IkbkbΔhep*) in male but not female mice. Gene profiling identifies sulfotransferase family 1E (*Sult1e1)*, which encodes a sulfotransferase E1 responsible for inactivation of estrogen, as a gene upregulated in NASH in both genders and most conspicuously in male *IkbkbΔhep* mice having worst NASH and lowest plasma estradiol levels. LXRα is enriched to LXRE on *Sult1e1* promoter in male WT and *IkbkbΔhep* mice with NASH, and a *Sult1e1* promoter activity is increased by LXRα and its ligand and augmented by expression of a S32A mutant of IκBα. These results demonstrate striking gender differences in regulation by IKKβ of high cholesterol saturated fat diet-induced metabolic changes including NASH and suggest hepatocyte IKKβ is protective in male due at least in part to its ability to repress LXR-induced *Sult1e1*. Our findings also raise a caution for systemic IKK inhibition for the treatment of NASH as it may exacerbate the disease in male patients.

## Introduction

Non-alcoholic fatty liver disease (NAFLD) including non-alcoholic steatohepatitis (NASH) is the most prevalent liver disease in the world and is commonly associated with obesity and insulin resistance (IR) [1–3]. Although the genetic and nutrient-deficient models such as that with choline and methionine deficient diet, have been widely used for experimental studies, it is well recognized that these models do not reflect a natural history or course of NAFLD/NASH seen in patients. To this end, different models have recently been developed which emphasize the common etiological backgrounds such as overnutrition [4] and a diet high in cholesterol and saturated fat (HCSF) [5–8].

Despite the obvious link between obesity, IR, and NASH, the precise mechanisms connecting these sequela are not fully understood. Inflammation is recognized as a potential causal factor in developing IR [9, 10], and activation of IκB kinase β (IKKβ) and c-Jun N-terminal kinase (JNK) are considered as critical effectors responsible for this causal link [11, 12]. The cell type in which IKKβ is activated, appears important in inducing IR in different organ sites. For instance, IKKβ in hepatocytes mediates diet-induced hepatic IR while that in myeloid cells is responsible for global IR [13]. Activation of IKKβ associated with endoplasmic reticulum (ER) stress in hypothalamus is recently shown to link overnutrition to suppressed insulin and leptin signaling in this tissue, leading to energy imbalance, obesity, and glucose intolerance [14]. Thus, these cell-type specific regulations by IKKβ appear to produce regional and systemic effects on energy homeostasis and insulin sensitivity culminating to obesity-associated IR. However, how these site-specific regulations actually relate to the pathogenesis of NASH is not known.

Another outstanding question is the mechanism of the gender effect on diet-induced obesity, IR, and NASH. Human and animal studies point to the importance of estrogen in energy and metabolic homeostasis. Postmenopausal women are susceptible to visceral obesity, IR and type 2 diabetes [15], and the treatment with 17β estradiol or conjugated estrogens attenuates these complications [16, 17]. In fact, the incidence of diabetes among postmenopausal women with hormone replacement therapy is significantly lower than those without [18] while the prevalence of NAFLD is higher in women than men at the age of 55 years or older without the therapy [19]. Conversely, among patients with persistent and unexplained elevations of serum transaminases at the average age of 46 years, many of whom had NAFLD and NASH, female gender was an independent factor associated with minimal liver lesions, suggesting that NAFLD/NASH is less severe in female patients before menopause [20]. However, how the gender affects the regulatory role of IKKβ in diet-induced obesity, IR and NASH, is completely unknown.

In the present study, we have examined the effects of hepatocyte or myeloid specific IKKβ deficiency on obesity, IR, and NAFLD/NASH induced by 20 wk feeding of a Western diet high in cholesterol and saturated fat diet (HCFD) in both male and female mice. Indeed, the gender has unique roles in influencing how IKKβ deficiency in these two cell types affects the metabolic complications. Further, we identify the potential role of sulfotransferase 1 (*Sult1e1*) in increased susceptibility for NASH in males particularly due to hepatocyte IKKβ deficiency.

## Materials and methods

### Animal experiments

Female and male C57BL/6J mice lacking IKKβ in hepatocytes (*IkbkbΔhep*) or myeloid cells (*IkbkbΔmye*) [13] were obtained from Dr. Michael Karin (Department of Pharmacology and Cancer Center University of California, San Diego, CA). C57BL/6/129 [21] and C57BL/6 [22] Cre-recombinase transgenic mouse lines, respectively, were used to generate myeloid- and hepatocyte-specific deleted IKKβ mice. These genetic deletions were confirmed to eliminate IKKβ expression in either all hepatocytes (*IkbkbΔhep*) or 80–90% of macrophages and neutrophils (*IkbkbΔmye*) [13]. IKKβ^F/F^ mice without hepatocyte or myeloid specific expression of Cre recombinase were used as wild type mice (Wt). All animals were treated in strict accordance with the recommendations in the *Guide for Care and Use of Laboratory Animals* of the National Institutes of Health. The protocol was approved by the Institutional Animal Care and Use Committee of the University of Southern California (Protocol Number: 10931). A HCFD diet used contained 37.6% calories (Cal) from fat (Lard:20.9% Cal), 16.9% Cal from protein (casein, L-Cystine), 45.5% Cal from carbohydrate (Sucrose, Cornstarch, Dextrose), Cholesterol 1% (w/w), vitamins and minerals (catalogue #180529, Dyets Inc., Bethlehem, PA). Standard rodent chow (control diet) used contained 20.8% Cal from fat, 22.1% Cal from protein, 57.1% Cal from carbohydrate (catalogue #S3888, Bio-Serv, Flemington, NJ). Mice were housed in a specific pathogen-free facility (12 hours light/dark cycle) and were fed either standard rodent chow or HCFD from 2.5 months of age for 20 wk or 12 months by the Animal Core of the Southern California Research Center for ALPD and Cirrhosis.

### Blood tests, glucose tolerance, and insulin sensitivity tests

Plasma glucose levels were determined by the GM7 Analyzer (Analox Instruments, Lunenburg, MA). Plasma ALT, insulin and adiponectin concentrations were measured by ELISA kits (ALT: Sigma Diagnostic, St. Louis, MO, insulin: Shibayagi Co., Gunma, Japan, adiponectin: ALPCO Diagnostics, Salem, NH, respectively). Mouse Estradiol in the plasma was determined by the Hormone Assay Core of the Vanderbilt Diabetes Center using a Double Antibody 17β- Estradiol RIA kit (MP Biomedicals, LLC Cat. # 07–138102). The assay was modified to improve the sensitivity to 1 pg /ml. The inter assay coefficient of variation was 10 and 6% at Mean = 4.5 and Mean = 22 pg/ml, respectively. After overnight fasting, glucose (1.5g/kg) or insulin (1 U/kg) was injected intraperitoneally, and blood glucose was monitored with Precision-Xtra strips (Medisense Products, Bedford, MA) at 0, 15, 30, 60, 120 min as previously performed [4].

### Histology and immunostaining

Paraffin-embedded sections were stained with hematoxylin and eosin, and histology was blindly scored for steatosis, inflammation, necrosis and lipo-granuloma by Morphology Core of the Southern California Research Center for ALPD and Cirrhosis. Liver fibrosis was assessed by reticulin staining and Sirius red staining. Formalin fixed sections of epididymal fat were immunostained for tumor necrosis factor-α (TNFα) F4/80, and CD68 using goat polyclonal (catalogue #AF-410, R&D Systems. Minneapolis, MN), rat monoclonal antibody (catalogue #MCA497, AbD Serotec/Bio-Rad, Morpho Sys, UKLTD, Oxford, UK), and rabbit polyclonal antibody (catalogue #sc-9139, Santa Cruz Biotech Inc, Santa Cruz, CA), respectively. Liver histology scoring was blindly evaluated by a subspecialty gastrointestinal pathologist as follows. Both micro-fat and macro-fat were individually scored according to the following scale: “0”: no fat; “1”: fat in up to 25% of the hepatocytes, “2”: up to 50%, “3”: up to 75%, and “4”: greater than 75%. The micro-fat and macro-fat scores were summed to determine the combined fat-micro, macro score. Inflammation, necrosis, lipogranuloma, and reticulin (fibrosis) were individually scored according to the following scale: “0”: none; “1”: minimal, “2”: mild, “3”: moderate, and “4”: severe (extensive). The inflammation and necrosis scores were summed to determine the combined inflammation/necrosis score. Fat histology scoring of inflammation in white adipose tissue (WAT) was also blindly evaluated by a subspecialty gastrointestinal pathologist according to the following scale: “0”: no inflammatory foci; “1”: minimal, “2”: mild, “3”: moderate, and “4”: severe (extensive) inflammatory foci.

### Immunoblot analysis

Liver tissues were homogenized in RIPA buffer (PBS, pH 7.4, 1% Nonidet P-40, 0.5% sodium deoxycholate, 0.1% SDS and complete protease inhibitor mixture). The protein extracts were resolved on a 10% GEL (SDS-PAGE), transferred onto a nitrocellulose membrane, then incubated with primary antibodies followed by incubation with horseradish peroxidase-conjugated secondary antibodies. The antigen-antibody complexes were visualized by the enhanced chemiluminescence detection system (Thermo Scientific, Rockford, IL). Antibodies against AMP kinase (AMPK), p-AMPK (Thr 172), JNK, p-JNK (catalogue #2532, #2531, #9252, #4668, respectively) were obtained from Cell Signaling Technology Inc. (Danvers, MA), nuclear form of sterol regulatory element binding protein-1c (nSREBP-1) (C-2) (catalogue #sc-366) was from Santa Cruz Biotech Inc., peroxisome proliferator-activated receptor-δ (PPARδ) antibody (catalogue #600-401-420) was purchased from Rockland Inc.(Gilbertsville, PA). Anti-β-actin antibody (catalogue #A1978) was obtained from Sigma-Aldrich (St. Louis, MO).

### Real-time polymerase chain reaction and microarray analysis

Total RNA was extracted using TRIzol reagent by Invitrogen (Carlsbad, CA). Total RNA was extracted from livers and white adipose tissue (WAT) using TRIzol reagent by Invitrogen. For real time polymerase chain reaction (PCR) analysis for PPARα, PPARδ, PPARγ, adiponectin, α1(I) procollagen, transforming growth factor-β (TGFβ1), CD68, TNFα, monocyte chemotactic protein-1 (MCP-1), Nos2, Arg-1 (arginase-1), Ym/Chi3l3, *Sult1e1*, osteoactivin (Gpnmb), the SYBR Green technique (Applied Biosystems, Foster City, CA) was used. Each Ct value was normalized by the GAPDH. The PCR primers used for these genes are as follows: 5’-ACGATGCTGTCCTCCTTGATG and 5’-GTGTGATAAAGCCATTGCCGT (PPARα); 5’-GCTGCTGCAGAAGATGGCA and 5’-CACTGCATCATCTGGGCATG (PPARδ); 5’-CCATTCTGGCCCACCAAC and 5’-AATGCGAGTGGTCTTCCATCA (PPARγ); 5’-AGAGATGGCACTCCTGGAGAGAA and 5’-CAACATCTCCTGTCTCACCCTTA (adiponectin); 5’-GCATGGCCAAGAAGACATCC and 5’-CCTCGGGTTTCCACGTCTC (α1(I) procollagen); 5’-TTGCTTCAGCTCCACAGAGA and 5’-TGGTTGTAGAGGGCAAGGAC (TGFβ1); 5’-CCAATTCAGGGTGGAAGAAA and 5’-TTGCATTTCCACAGCAGAAG (CD68); 5’-TACAGGCTTGTCACTCGAATT and 5’-ATGAGCACAGAAAGCATGATC (TNFα); 5’-CCCAATGAGTAGGCTGGAGA and 5’-TCTGGACCCATTCCTTCTTG (MCP-1); 5’-GCCATTGCACAACTCTTTTC and 5’-GGAGAGGAGACTTCACAG (interleukin-6, or IL-6); 5’-CACCAAGCTGAACTTGAGCGA and 5’-CCATAGGAAAAGACTGCACCGA (Nos2); 5’-CTCCAAGCCAAAGTCCTTAGAG and 5’-AGGAGCTGTCATTAGGGACATC (Arg-1); 5’-AGGAAGCCCTCCTAAGGACA and 5’-ACGTCAATGATTCCTGCTCC (chitinase 3-like 3, or Ym1/Chi3l3); 5’-CTTCCAGCATCATTTTGGGAAAAG and 5’-TGGATTGTTCTTCATCTC (*Sult1e1*); 5’-TTCACTGTGACCTGCAAAGG AND 5’-CAGTAGGTGCCAGACCCATT (Gpnmb). Microarray analysis was performed on liver RNA samples using Mouse Genome 430 2.0 arrays (Affymetrix, Santa Clara, CA) at the USC/CHLA Genome Core Laboratory.

### *Sult1e1* activity, promoter and ChIP analysis

SULT1E1 activity was determined using ^35^S-phosphoadenosine phosphosulfate (PerkinElmer, Wellesley, MA) as previously described [23]. Chromatin isolated from liver tissues were analyzed for LXRα enrichment to a putative DR-4 of *Sult1e1* gene (nt-121/-142: ataAGGTCAagttTGCTCActc) by chromatin immunoprecipitation analysis (ChIP) and qPCR using the primers, 5’-CCAAAGGGGAGAAACAGCTG-3’ and 5’-GAGAAGGAGGCAGAGACTC- GGGGA as previously described [23]. *Sult1e1* promoter activity was analyzed using tk- *Sult1e1*/DR4 promoter-luciferase in HepG2 cells with or without expression of LXRα and/or a dominant negative mutant (S32A) of IκBα, or treatment with TNFα. This tk-DR4 (mSult1e1)-Luciferase construct is a heterologous reporter generated by inserting three copies of the DR4 (direct repeat spaced by 4 nucleotides) type nuclear receptor response element into the thymidine kinase (tk)-Luciferase reporter gene; the DR4 sequence is (nt -121) ataAGGTCAagttTGCTACatc (nt -142), as previously described [23],

### Data analysis

The numerical data were expressed as the means ±S.E.M. unless otherwise noted. ANOVA followed by post-hoc Tukey test was performed for multiple comparisons denoting significant differences with †p<0.05 and ††p<0.01, when appropriate. Student’s *t-*test was performed to assess the statistical significance between two sets of data, as appropriate. *p* values less than 0.05 were considered significant as denoted by the asterisk.

## Results

### Obesity and visceral adiposity induced by HCFD are attenuated by myeloid IKKβ deficiency in female but not male mice and aggravated by hepatocyte IKKβ deficiency in males but not females

**Fig 1:**
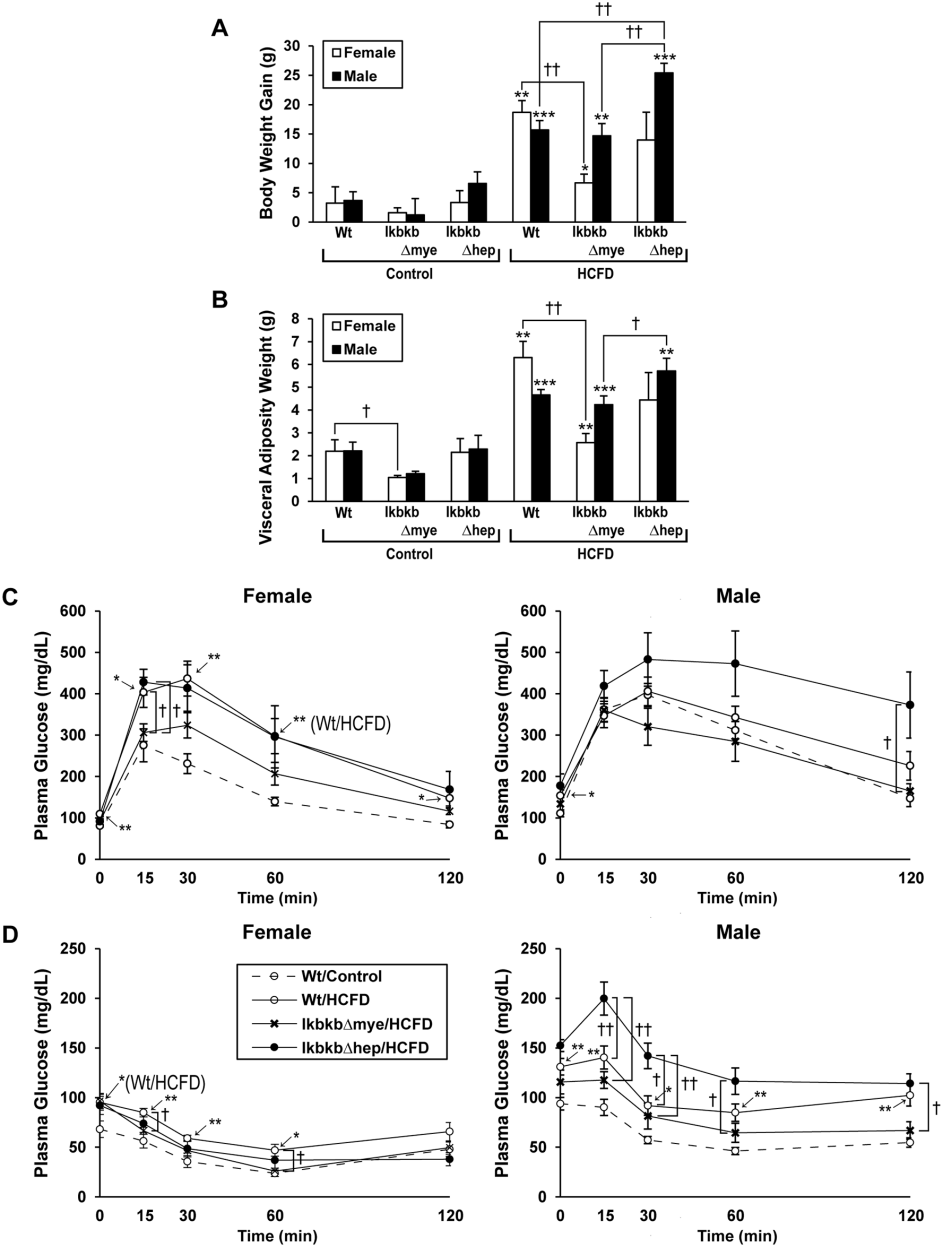
Weight gain, visceral adiposity, and plasma glucose. Wild type (Wt), myeloid IKKβ deficient (*IkbkbΔmye*), or hepatocyte IKKβ deficient (*IkbkbΔhep*) mice in both genders, were fed regular chow (Control) or high cholesterol and saturated fat diet (HCFD) from 2.5 months of age for 20 weeks, and body weight gain (A) and visceral adiposity weight (B) were determined. Visceral adiposity was determined by summing the individual measures of epidydimal/gonadal fat, mesenteric fat, and retroperitoneal fat. Glucose tolerance (C) and insulin sensitivity (D) tests were also performed after overnight fast. *p<0.05, **p<0.01, ***p<0.001 compared to Control diet within gender and within genotype by t-test. †p<0.05, ††p<0.01 compared to other genotype within gender and within diet by ANOVA and post-hoc Tukey test.

Feeding HCFD for 20 wk causes significant obesity in both male and female Wt mice as compared to chow-fed (control) Wt mice (Fig 1A) despite comparable daily caloric intake of two diets (mean ±S.D.: 17.4±2.5 for control males vs. 16.8±3.6 Cal/day for HCFD-fed males;14.2±4.6 for control females vs. 13.2±4.7 Cal/day for HCFD-fed females). IKKβ deficiency in myeloid cells (*IkbkbΔmye*) almost completely prevents obesity in females but has no effects in males (Fig 1A). In contrast, IKKβ deficiency in hepatocytes (*IkbkbΔhep*) aggravates HCFD-induced obesity in males but not in females (Fig 1A). The total weight of visceral adipose tissues shows similar gender-specific changes: prevention of a HCFD-induced increase in female but not male *IkbkbΔmye* mice and aggravation of this parameter in male but not female *IkbkbΔhep* mice (Fig 1B). Female *IkbkbΔmye* mice on chow diet also showed decreased visceral adiposity compared to their Wt counterparts.

### HCFD-induced hyperglycemia and global IR are attenuated by myeloid IKKβ deficiency in females but aggravated in male mice by hepatocyte IKKβ deficiency

HCFD feeding increases the fasting glucose levels in both male (mean ±S.D.: 100±21 vs. 142±32 mg/dl, p<0.005) and female (75±10 vs. 105±16 mg/dl, p<0.005) WT mice. These hyperglycemias tend to be attenuated in male (121±34 mg/dl) and female (93±22 mg/dl) *IkbkbΔmye* mice. Fasting hyperglycemia tends to be aggravated in male (158±22 mg/dl) but not female (92±23 mg/dl) *IkbkbΔhep* mice. Fasting insulin levels are significantly elevated in HCFD-fed male Wt mice compared to chow-fed male Wt mice (mean ±S.D.: 0.96±0.5 vs. 0.51±0.45 ng/ml, p<0.05). This hyperinsulinemia tends to be corrected in *IkbkbΔmye* (0.64±0.42 ng/ml) but not in *IkbkbΔhep* (0.97±0.59) mice. In females, plasma insulin levels are not different among different genotypes and dietary groups. Glucose tolerance test reveals HCFD-fed female Wt mice are glucose intolerant as compared to chow-fed Wt mice, and this effect is attenuated in *IkbkbΔmye* mice (Fig 1C). Insulin sensitivity test demonstrates insulin resistance in both female and male Wt mice fed HCFD. This effect is largely corrected in female and partially ameliorated in male IkbkbΔmye mice (Fig 1D). On the other hand, insulin resistance is worsened in male but not female *IkbkbΔhep* mice (Fig 1D). In summary, HCFD feeding causes obesity, increased visceral adiposity, hyperglycemia, reduced glucose tolerance and insulin resistance in both genders. IKKβ deficiency in myeloid cells clearly largely corrected these metabolic effects in females but only partially in males. On the contrary, IKKβ deficiency in hepatocytes aggravates most of these effects in males but not in females.

### NASH is attenuated in *IkbkbΔmye* mice in both genders but aggravated in male but not female *IkbkbΔhep* mice

**Fig 2:**
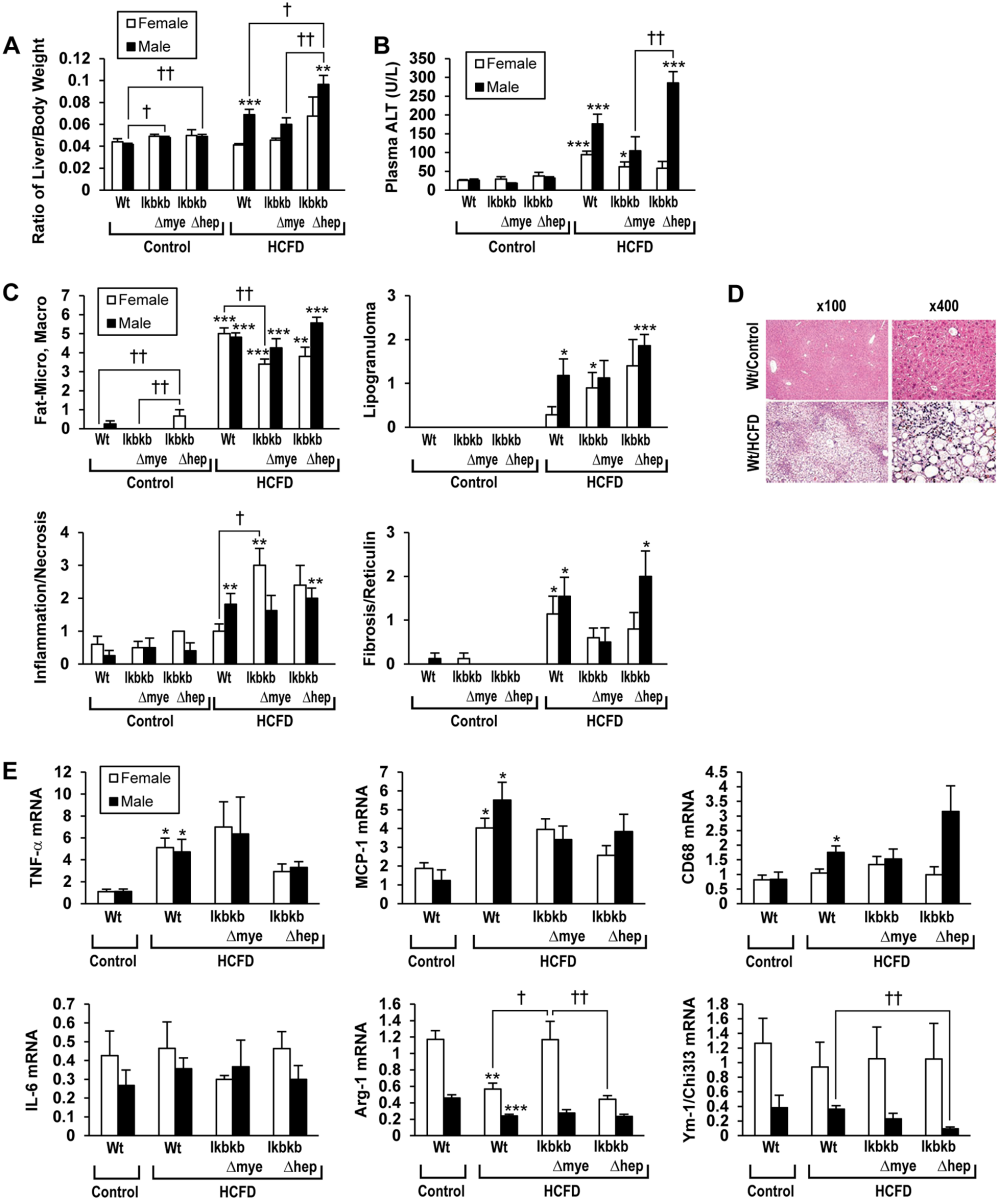
HCFD feeding produces NASH in Wt mice. Wild type (Wt), myeloid IKKβ deficient (*IkbkbΔmye*), or hepatocyte IKKβ deficient (*IkbkbΔhep*) mice in both genders, were fed regular chow (Control) or high cholesterol and saturated fat diet (HCFD) from 2.5 months of age for 20 weeks. (A) Hepatomegaly was assessed by the ratio of liver weight over body weight. (B) Plasma ALT was measured. (C) Liver histological grading was determined for micro- and macro-vesicular steatosis, necroinflammation and lipogranuloma. Liver fibrosis was assessed by analysis of reticulin staining. (D) Representative microphotograph of typical NASH induced in a HCFD-fed Wt male mouse as compared to normal liver histology of a chow-fed male Wt mouse (Wt/Control, upper panels). (E) mRNA expressions in the liver of M1 genes (TNFα, MCP-1), macrophage marker CD68, IL-6, and M2 genes (Arg-1 and Ym1/Ch3l3). *p<0.05, **p<0.01, ***p<0.001 compared to Control diet within gender and within genotype. †p<0.05, ††p<0.01 compared to other genotype within gender and within diet.

HCFD feeding results in hepatomegaly as assessed by the ratio of liver to body weight in male but not female Wt mice. This effect is prevented in male *IkbkbΔmye* mice but worsened in *IkbkbΔhep* mice (Fig 2A). Interestingly, both chow-fed male *IkbkbΔmye* and *IkbkbΔhep* mice have increased ratio of liver to body weight compared with Wt mice of the same gender. Plasma ALT levels are increased by HCFD feeding in Wt mice, and a higher elevation is observed in male Wt mice than females (Fig 2B). These elevations associated with HCFD feeding, are attenuated in *IkbkbΔmye* mice in both genders (Fig 2B). Histologically, HCFD feeding causes macro and micro-vesicular steatosis, inflammation and necrosis in Wt mice in both genders (Fig 2C). A typical histology of NASH seen in male Wt mice fed HCFD is shown in Fig 2D as compared to normal histology in Wt control. The pathological changes of macro and micro-vesicular steatosis induced by HCFD are attenuated in *IkbkbΔmye* female mice (Fig 2C). Real-time PCR results demonstrate that HCFD-induced NASH is associated with increased expression of M1 macrophage activation genes such as TNFα and MCP-1 and decreased expression of the M2 alternative activation gene arginase-1 (*Arg-1*) [24] in both genders (Fig 2E). The *IkbkbΔmye* genotype rescues the suppressive effect of HCFD on Arg-1 in female but not in male mice. In fact, the expression of both Arg-1 and another M2 gene *Ym-1/Chi3l3*, which encodes a heparin-binding lectin or chitinase 3-like 3 [25], are significantly lower in male mice in all genotypes (Fig 2E). Expression of CD68, the marker for macrophages, is increased significantly in male Wt mice fed HCFD and this tends to increase further in HCFD-fed *IkbkbΔhep* mice. IL-6 expression tends to be lower in males than in females in all dietary and genotype groups, but is not affected by HCFD or genotypes.

**Fig 3:**
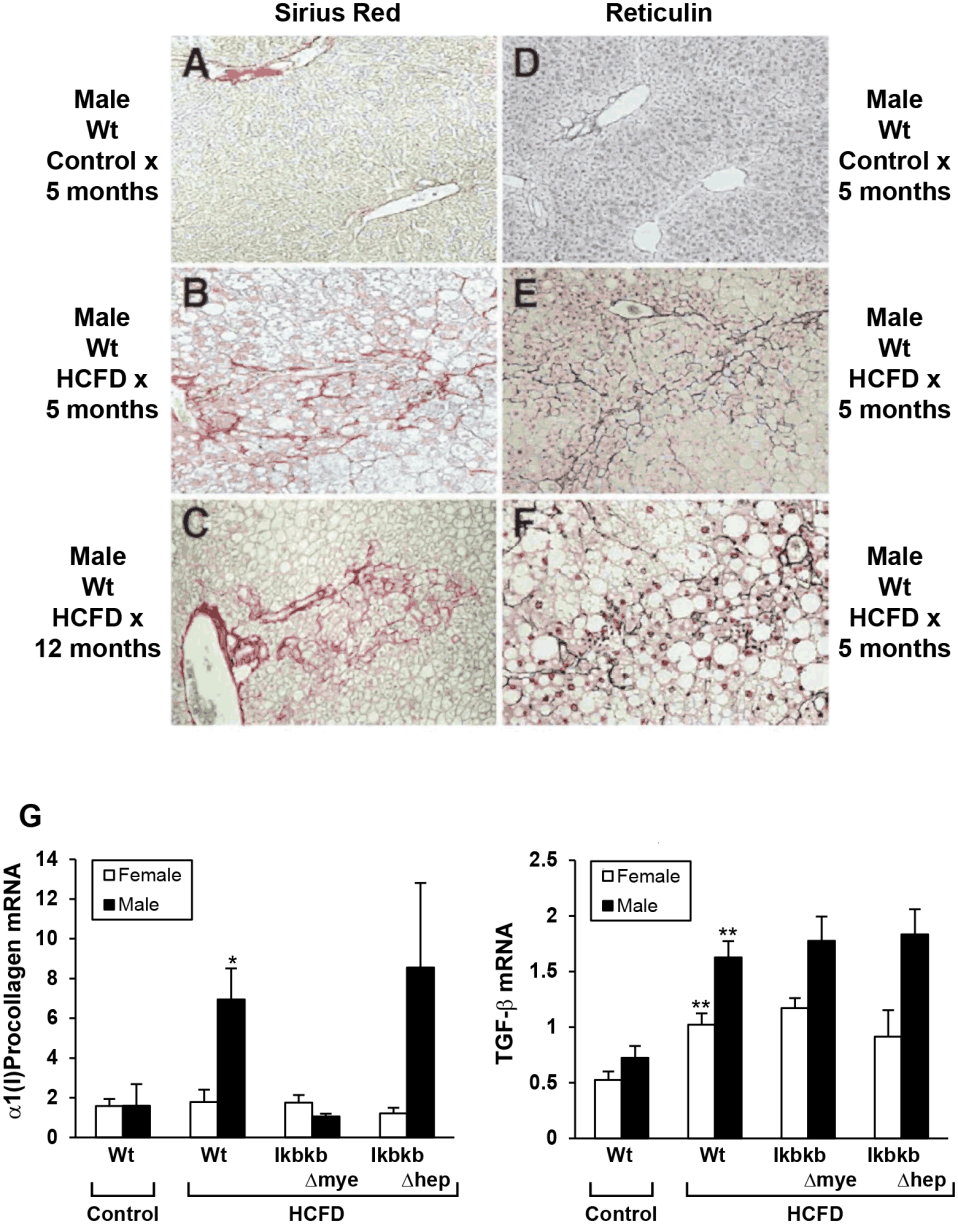
Liver fibrosis induced by HCFD. Liver fibrosis as assessed by Sirius red staining (A-C, representative microphotographs), reticulin staining (D-F, representative microphotographs), or real-time PCR for α1(l)procollagen and TGFβ1 mRNA (G). Wild type (Wt), myeloid IKKβ deficient (*IkbkbΔmye*), or hepatocyte IKKβ deficient (*IkbkbΔhep*) mice were fed regular chow (Control) or high cholesterol and saturated fat diet (HCFD) from 2.5 months of age for 20 weeks (A, B, and D-F) or 12 months (C). (A, C, D, E at x169 magnification; B and F at x338 magnification.) *p<0.05, **p<0.01 compared to Control diet within gender and within genotype.

HCFD feeding also induces liver fibrosis in Wt and *IkbkbΔhep* mice as demonstrated by Sirius red and reticulin staining (Fig 3B–3F and data not shown). Pericellular and perisinusoidal fibrosis are commonly observed. The fibrosis score as rated by reticulin staining reveals less fibrosis in female Wt mice; attenuation in *IkbkbΔmye* mice in both genders; and aggravation in male but not female *IkbkbΔhep* mice (Fig 2C). Minimal staining by Sirius red or reticulin are noted in liver sections of a male Wt mouse fed chow for 5 months (Fig 3A and 3D). Sirius red staining of liver section of a male Wt mouse fed HCFD for 5 months shows collagen deposition forming bridges from the portal area (left) to the central vein (right) (Fig 3B). Sirius red staining of a male Wt mouse fed HCFD for 12 months reveals the portal to central vein bridging fibrosis (Fig 3C). Reticulin staining of liver section from a male Wt mouse fed HCFD for 5 months depicts pericellular “Chicken wire” pattern of fibrosis typical of NASH (Fig 3E). Another liver section of 5 months HCFD-fed Wt mouse stained for reticulin shows pericellular fibrosis with clusters of PMNs at a higher magnification (Fig 3F). Expression of α1(I)procollagen and the pro-fibrogenic cytokine, TGFβ1, mRNAs correlate with fibrotic changes. Increased hepatic expression of α1(l)procollagen mRNA by 5-month HCFD feeding is noted for male Wt mice, and tends to be attenuated in male *IkbkbΔmye* mice, but further enhanced in male *IkbkbΔhep* mice. Expression of TGFβ1 is increased by 5-month HCFD in Wt mice of both genders, and not affected by genotype on this diet (Fig 3G). For a subset of Wt male animals, we continued HCFD feeding for 12 months to determine a long-term effect on liver fibrosis. Bridging liver fibrosis is evident in most of 12 month HCFD-fed mice (Fig 3C).

### Enhanced lipogenic regulation by HCFD

**Fig 4:**
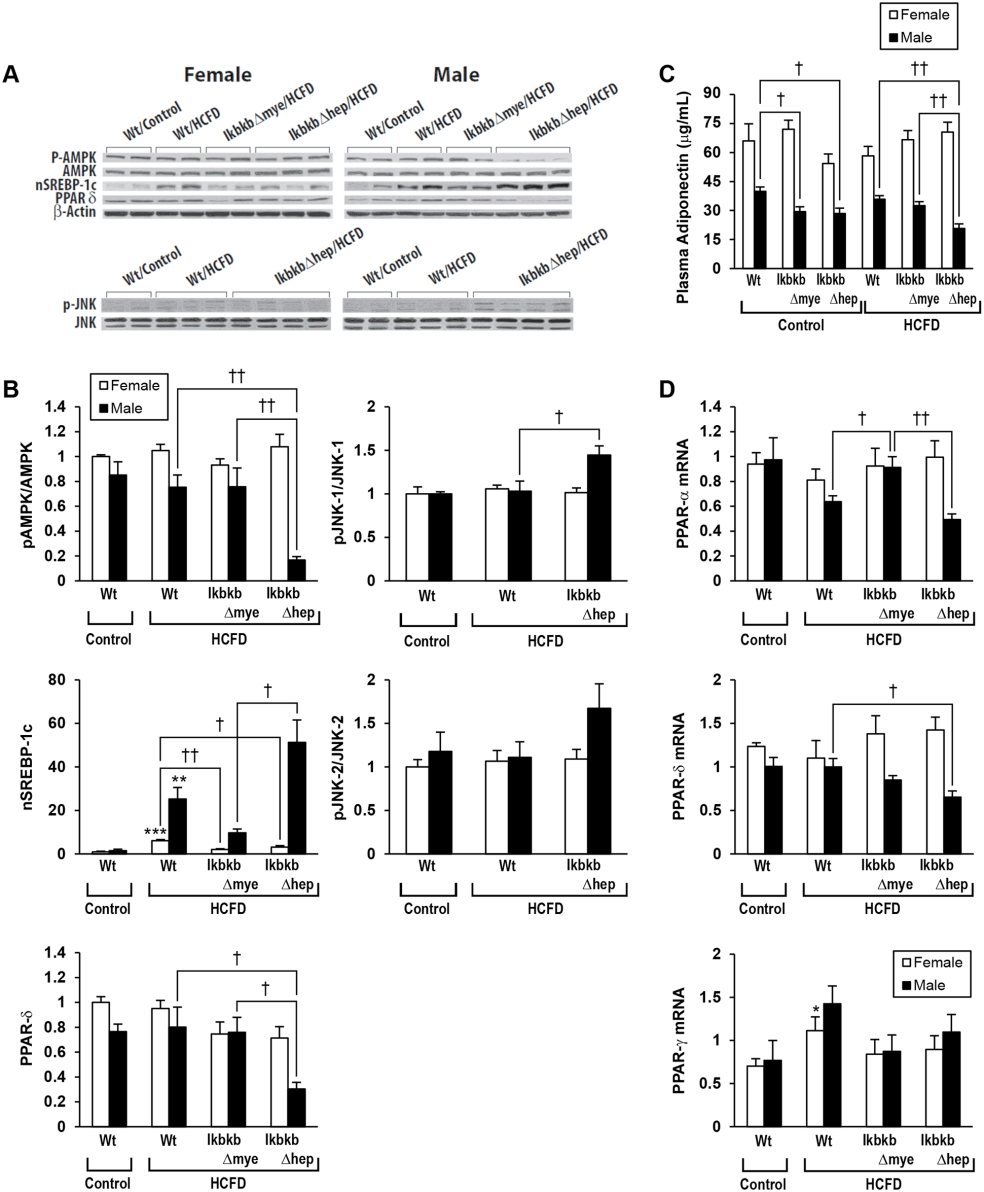
Upregulation of lipogenic genes and down-regulation of lipolytic genes associated with NASH induction and aggravation. Wild type (Wt), myeloid IKKβ deficient (*IkbkbΔmye*), or hepatocyte IKKβ deficient (*IkbkbΔhep*) mice in both genders, were fed regular chow (Control) or high cholesterol and saturated fat diet (HCFD) from 2.5 months of age for 20 weeks. (A) Immunoblotting of whole liver lysate and (B) densitometric analysis of pAMPK/AMPK, nSREBP-1c, PPARδ, pJNK-1/JNK-1, and pJNK-2/JNK-2. (C) Plasma adiponectin levels measured by ELISA. (D) Real-time PCR results of PPAR α, δ, and γ genes. *p<0.05, **p<0.01, ***p<0.001 compared to Control diet within gender and within genotype. †p<0.05, ††p<0.01 compared to other genotype within gender and within diet.

HCFD feeding increases the hepatic levels of a nuclear (activated) form of SREBP-1c (nSREBP-1c), a potent lipogenic transcription factor, in Wt mice in both genders but more conspicuously in males (Fig 4A and 4B). HCFD does not affect the levels of p-AMPK and PPARδ, both considered as anti-lipogenic and lipolytic regulators [26, 27], in male and female Wt mice (Fig 4A and 4B). Expression of mRNA for PPARγ, another lipogenic transcription factor, is increased in HCFD-fed Wt mice in females and tends to be increased in males, while that for PPARα, another lipolytic transcription factor, tends to be reduced in male HCFD-fed Wt mice (Fig 4D). In *IkbkbΔmye* mice, the increase in nSREBP-1c levels caused by HCFD is attenuated in females (Fig 4A and 4B), and PPARγ mRNA tends to be reduced in males (Fig 4D). In addition, PPARα mRNA is normalized in male *IkbkbΔmye* mice-fed HCFD (Fig 4D). More drastic changes are seen in HCFD-fed male *IkbkbΔhep* mice with aggravated NASH. Firstly, p-AMPK and PPARδ protein levels are severely reduced, nSREBP-1c is most upregulated, and p-JNK1 levels are clearly elevated (Fig 4A and 4B) while p-JNK2 tends to be increased. In addition, PPARα mRNA tends to be further decreased in HCFD-fed male *IkbkbΔhep* mice as compared to HCFD-fed Wt male mice (Fig 4D). Plasma levels of adiponectin, an adipokine which serves as an activator of p-AMPK, are significantly lower in males than in females within genotype and within diet (Fig 4C). In male *IkbkbΔhep* mice-fed HCFD, the level is most reduced compared to other genotypes fed HCFD (Fig 4C). Thus, these results demonstrate that HCFD-induced NASH in male Wt mice is associated with increased expression of lipogenic regulators (nSREBP-1c, PPARγ) and reduced lipolytic factor PPARα. These changes are reversed by the IKKβ deficiency in myeloid cells in both genders and aggravated only in males by the IKKβ deficiency in hepatocytes. Reduced AMPK activation perhaps at least partially due to decreased plasma adiponectin levels and conversely increased JNK1/2 activation are also evident in aggravated NASH in male *IkbkbΔhep* mice.

### WAT inflammation correlates with HCFD-induced NASH

**Fig 5:**
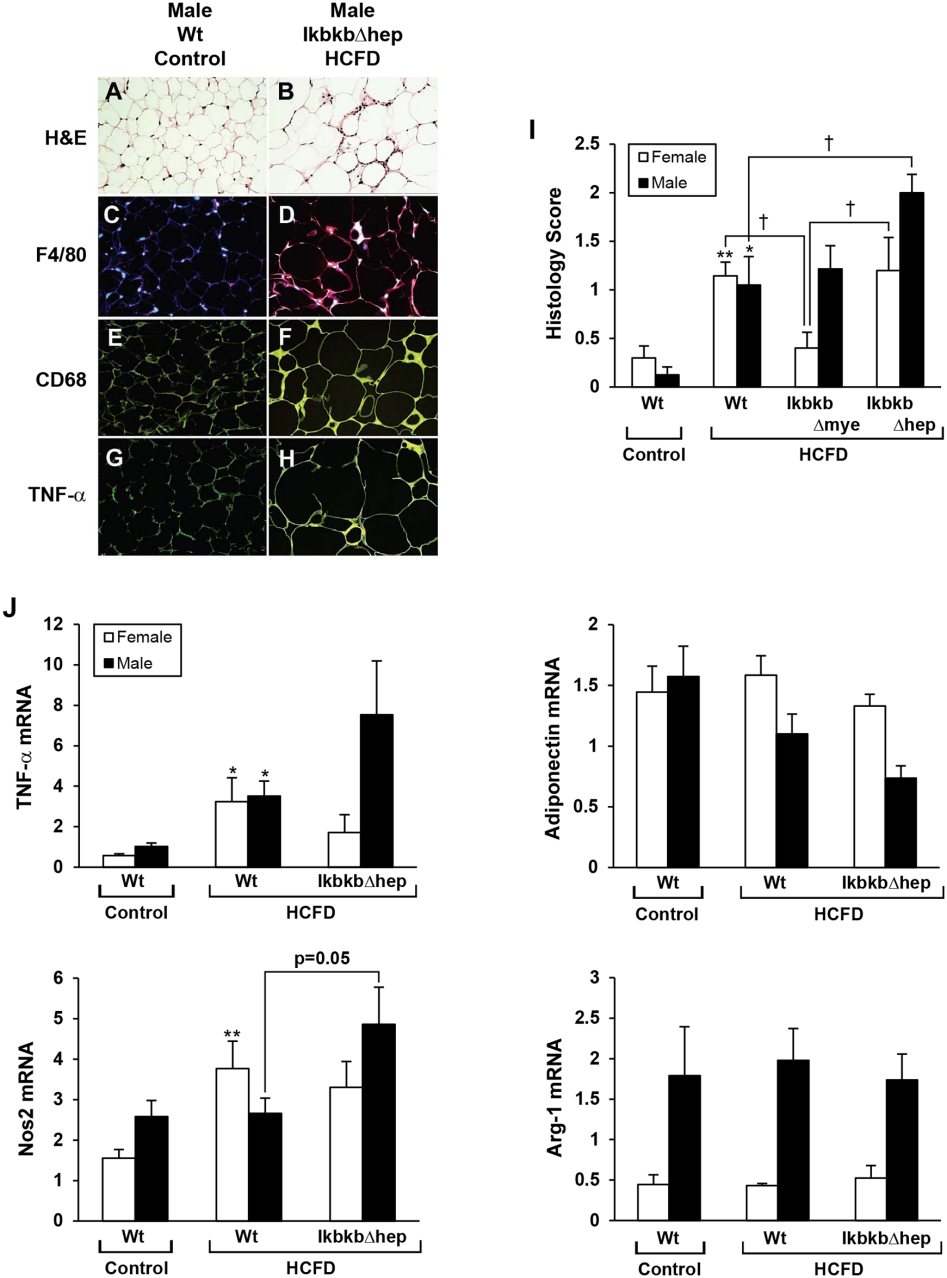
Inflammation in epididymal fat tissue induced by HCFD in Wt mice of both genders and aggravated by *IkbkbΔhep* in males. (A), (C), (E) and (G) are representative microphotographs from a male Wt mouse fed control diet from 2.5 months of age for 20 weeks as opposed to (B), (D), (F) and (H) from a male *IkbkbΔhep* mouse fed HCFD with aggravated NASH. Sections stained with H&E (x260). (B and D) Immunofluorescent staining for the macrophage marker F4/80 (red) (x260). (E and F) Immunofluorescent staining for another macrophage marker CD 68 (green) (x260). (G and H) Immunofluorescent staining for TNFα (green) (x260). (I) Fat histology scoring of inflammation in white adipose tissue was determined by assessment of inflammatory foci. (J) Real-time PCR for TNFα, Nos2, adiponectin, and the M2 gene Arg-1 in white adipose tissue. *p<0.05, **p<0.01 compared to Control diet within gender and within genotype. †p<0.05 compared to other genotype within gender and within diet.

WAT inflammation is suggested to be causally linked to obesity-associated IR [9, 10]. HCFD feeding increases the frequency of inflammatory foci in WAT of both female and male Wt mice (Fig 5I) as detected by H&E staining (Fig 5B) and confirmed by immunohistochemistry for F4/80 (Fig 5D) and CD68 (Fig 5F) as compared to respective staining in WAT from chow-fed Wt mice (Fig 5A, 5C and 5E). TNFα immunostaining in WAT is also increased by HCFD feeding (Fig 5H) as compared to chow-fed Wt (Fig 5G). Real-time PCR analysis demonstrates increased TNFα expression in WAT of both male and female HCFD-fed Wt mice, and a tendency to further increment in this gene expression in male *IkbkbΔhep* mice (Fig 5J). Conversely, WAT adiponectin expression tends to be reduced by HCFD feeding in male but not female Wt mice and tends to further decrease in male *IkbkbΔhep* mice (Fig 5J), corroborating the reduced plasma adiponectin levels in this group (Fig 4C). *Nos2*, another M1 gene, is also upregulated particularly in WAT of male HCFD-fed *IkbkbΔhep* mice in agreement with most severe fat inflammation seen in these mice (Fig 5I). *Arg-1* expression in WAT is uniformly higher in male than female mice within genotype and within diet and is not affected by HCFD or IKKβ deficiency.

### Sulfotransferase family 1E (*Sult1e1*) and osteoactivin (*Gpnmb*) are differentially induced in male but not female *IkbkbΔhep* mice fed HCFD

**Fig 6:**
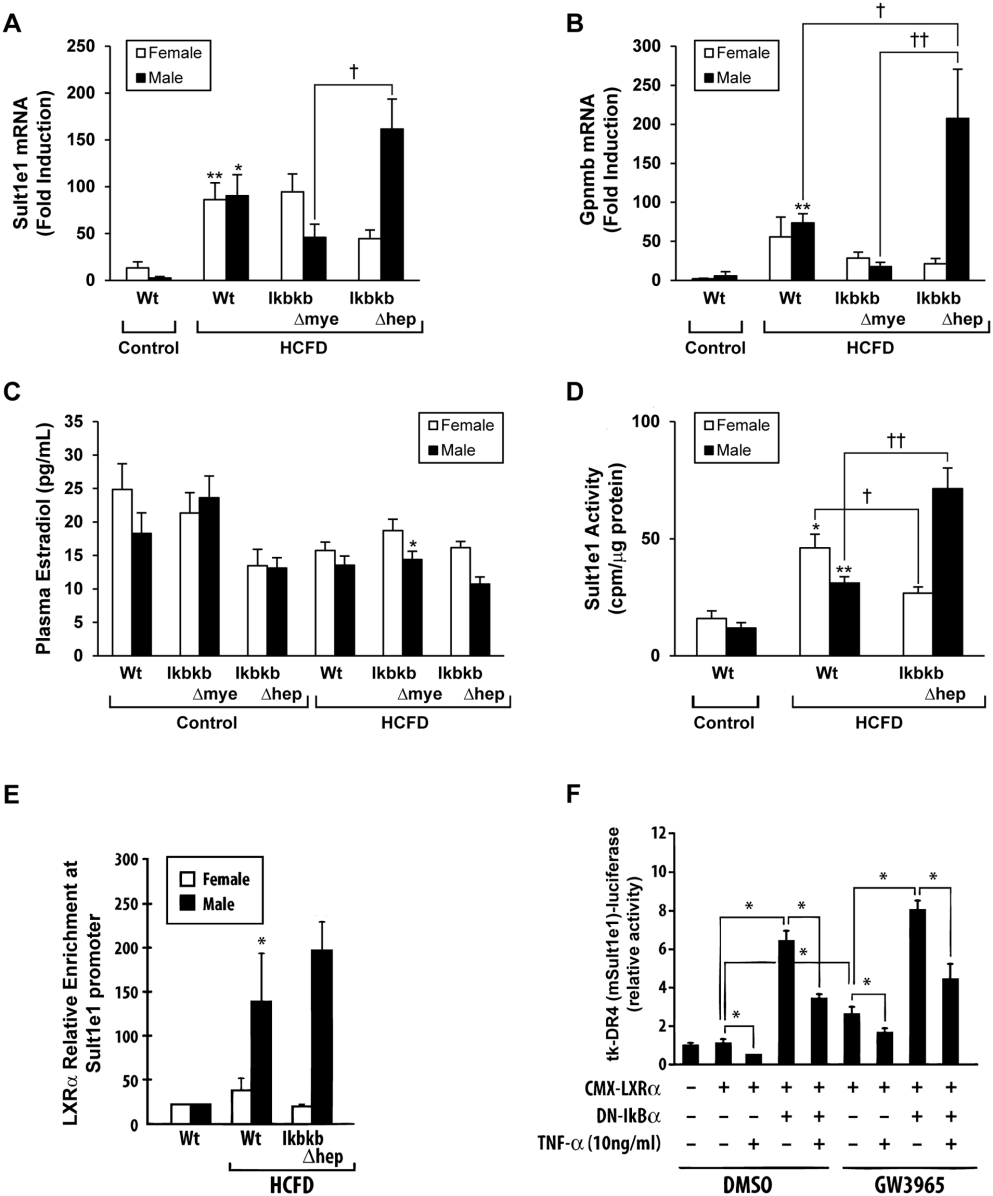
*Sult1e1* and *Gpnmb* are differentially up-regulated by *IkbkbΔhep* in males but not females fed HCFD. Wild type (Wt), myeloid IKKβ deficient (*IkbkbΔmye*), or hepatocyte IKKβ deficient (*IkbkbΔhep*) mice in both genders, were fed regular chow (Control) or high cholesterol and saturated fat diet (HCFD) from 2.5 months of age for 20 weeks (A-E). Real-time PCR of *Sult1e1* (A) and Gpnmb (B) expression. (C) Plasma estrogen levels assessed by double-antibody radioimmunoassay. (D) SULT1E1 activity assessed by sulfotransferase assay carried out as previously described [23]. (E) Chromatin isolated from liver tissues were analyzed for LXRα enrichment to a putative DR-4 of *Sult1e1* gene as previously described [23]. (F) *Sult1e1* promoter activity was analyzed using tk-DR4 (mSult1e1)-Luciferase construct in HepG2 cells with or without expression of LXRα and/or a dominant negative mutant (S32A) of IκBα, or treatment with TNFα. [(A-E) *p<0.05, **p<0.01 compared to Control diet within gender and within genotype. †p<0.05, ††p<0.01 compared to other genotype within gender and within diet. (F) *p<0.05 between the compared data.]

The hepatocyte IKKβ deficiency worsens HCFD-induced NASH in male but not female mice. To help understand this gender difference, we have performed microarray analysis for liver RNA samples from male and female Wt and *IkbkbΔhep* mice fed HCFD and determined genes which are differentially regulated by IKKβ deficiency in male but not female mice as compared to Wt mice. Table 1 shows partial lists of genes up-regulated or down-regulated by hepatocyte IKKβ deficiency in males. (Complete gene lists are available online as supplementary information, S1 and S2 Tables. In addition, the microarray data may be accessed online via the NIH GEO website by using accession number GSE99031.) Among the genes up-regulated, *Sult1e1* encodes sulfotransferase family E1 which is responsible for sulfation of estrogen, the first step in inactivation and metabolism of this hormone [28]. *Gpnmb* encodes osteoactivin, a TGF-β family of transmembrane glycoprotein which is expressed by macrophages and transformed cells and implicated in inflammation including liver injury caused by CCl4 [29] or choline-deficient diet [30], cell invasion and migration via MMP induction [30, 31]. Induction of *Sult1e1* by HCFD feeding tends to be attenuated in male *IkbkbΔmye* mice but further enhanced in male but not female *IkbkbΔhep* mice (Fig 6A). *Gpnmb* induced by HCFD in Wt mice tends to be suppressed in male *IkbkbΔmye* mice and accentuated ~3-fold in male but not female *IkbkbΔhep* mice (Fig 6B).

**Table 1:** Differentially regulated genes in male vs. female *IkbkbΔhep* mice-fed HCFD*.

Change in Males	Change in Females	Gene ID: Code	Gene Description
+2.1	-1.2	1053111: *Sult1e1*	Sulfotransferase family 1E
+1.73	-1.06	10574023: Mt1e	Methallothionein 1E
+1.65	1	10407281: Esm1	Endothelial cell specific molecule 1
+1.61	1	10578352: Fgl1	Fibrinogen-like 1
+1.60	-2.1	10538187: Gpnmb	Osteoactivin
+1.59	1	10551209: Cyp2b13	Cytochrome P450 2B13
+1.54	-1.53	10468239: Cyp17a1	Cytochrome P450 17A1
+1.52	-1.1	10357261: Macro	Macrophage receptor with collagenous structure
-3.7	1	10566326: Trim12	Tripartite motif-containing 12
-2.0	1	10497349: Sirpb1	Signal-regulatory protein beta 1
-1.98	1	10392839: Cd300e	CD300e antigen
-1.78	1	10351509: Fcgr4	Fc receptor, IgG, low affinity IV
-1.55	+1.1	10445953: Emr4	EGF-like molecule containing mucin-like, hormone receptor-like sequence 4
-1.53	+1.3	10562169:Hamp	Hepcidin

*Four RNA samples from wildtype mice-fed HCFD and *IkbkbΔhep* mice-fed HCFD in both genders were pooled respectively, analyzed by Affymetrix microarray, and compared against respective wildtype mouse samples in each gender.

Note: Liver tissue for microarray analysis was collected from mice fed HCFD for 20 weeks since 2.5 months of age.

SULT1E1 activity in the liver is increased by HCFD in both genders and further increased only in male *IkbkbΔhep* mice (Fig 6D). As predicted, the plasma estradiol levels tend to be reduced by HCFD feeding in both male and female Wt mice and further decreased in male but not female *IkbkbΔhep* mice (Fig 6C), suggesting up-regulation of *Sul1e1* and its activity are responsible for reduced estradiol levels. As estradiol is known to protect from IR and hepatic lipogenic regulation [32], these results suggest that heightened induction of *Sult1e1* by HCFD and IKKβ deficiency in male mice results in the lower circulating levels of estradiol and worsening of IR and NASH in this gender. *Sult1e1* induction observed in our model suggests a LXR-mediated mechanism as our diet is high in cholesterol and *Sult1e1* promoter is known to contain functional LXRE [23]. To test the involvement of LXRα in *Sult1e1* induction seen in the models, we performed ChIP analysis for recruitment of this nuclear hormone receptor to the *Sult1e1* promoter with a putative DR4 sequence for LXRE [27]. LXRα enrichment to the promoter is increased in the livers of male but not female Wt and *IkbkbΔhep* mice fed HCFD (Fig 6E). We next performed a transfection-reporter analysis with a DR4(m*Sult1e1*)-luciferase construct in HepG2 cells. Expression of LXRα alone does not increase the promoter activity (2^nd^ vs. 1^st^ bar, Fig 6F), but in the presence of the LXRα agonist GW3965, the activity is increased 3-fold (6^th^ bar). Expression of a dominant negative S32A mutant (DN) of IκBα which blocks NF-κB activation, causes a marked increase in the promoter activity (4^th^ bar) while the treatment with TNFα which activates NF-κB, inhibits it (3^rd^ bar) and attenuates the increase caused by DN-IκBα (5^th^ bar). Under the GW3965 treatment, similar patterns of the effects of DN-IκBα and/or TNFα are observed. These results demonstrate that the *Sult1e1* promoter containing a putative DR-4 for LXRE, is under positive regulation of LXRα and its agonist and negatively regulated by NF-κB. These findings suggest heightened induction of *Sult1e1* in the liver of male *IkbkbΔhep* mice, is due to a loss of the NF-κB mediated inhibition of the gene.

## Discussion

The present study demonstrates the gender-specific, differential regulatory roles of IKKβ in myeloid cells and hepatocytes in obesity, IR, and NASH induced by HCFD in mice as summarized in Table 2. Key findings can be summarized as follows. Firstly, HCFD feeding to adult C57Bl/6 mice results in a wide spectrum of metabolic syndrome as previously reported [33], including obesity, visceral adiposity, hyperglycemia, global IR and NASH, and these complications are generally more pronounced in males than females. Secondly, although myeloid IKKβ deficiency corrects obesity and visceral adiposity in female but not male mice, it ameliorates fasting hyperglycemia, global IR, and steatohepatitis in both genders. Thirdly, hepatocyte IKKβ deficiency aggravates all of these sequela in male, but not in female mice. These gender specific differences are closely associated with: 1) lower plasma adiponectin levels in males regardless of diet or genotype; 2) lower p-AMPK and higher nSRBP-1c in the livers of males fed HCFD compared to females; 3) marked reductions in plasma adiponectin, hepatic p-AMPK and PPARδ levels and conspicuous increases in nSREBP-1c and p-JNK1/2 in aggravated NASH in male hepatic IKKβ deficient mice fed HCFD; 4) lower hepatic M2 gene (Arg1 and Chi3l3) expression in males than females regardless of diet or genotype; 5) further repression of these M2 genes in aggravated NASH in male hepatic IKKβ deficient mice; 6) inflammation in WAT associated with HCFD-induced NASH which is ameliorated by myeloid IKKβ deficiency in females and aggravated by hepatic IKKβ deficiency in males.

**Table 2:** Summary of changes in key metabolic parameters*.

	Wt-fed HCFD		*IkbkbΔmye-fed HCFD*		*IkbkbΔhep-fed HCFD*	
	Female	Male	Female	Male	Female	Male
Obesity	**↑**	**↑**	**—**	**↑**	**↑**	**↑↑**
Visceral Adiposity	**↑**	**↑**	**—**	**↑**	**↑**	**↑↑**
Hyperglycemia	**↑**	**↑**	**—**	**—**	**↑**	**↑↑**
Global IR	**↑**	**↑**	**—**	**↑**	**↑**	**↑↑**
NASH	**↑**	**↑**	**—**	**—**	**↑**	**↑↑**
Lipogenic genes	**↑**	**↑**	**—**	**↑**	**↑**	**↑↑**
Lipolytic genes	**—**	**↓**	**—**	**—**	**—**	**↓↓**
p-JNK1/2	**—**	**—**	**—**	**—**	**—**	**↑**
Liver M1>M2	**↑**	**↑**	**↑**	**↑**	**↑**	**↑↑**
Adiponectin	**—**	**↓**	**—**	**—**	**—**	**↓↓**
WAT Inflamm.	**↑**	**↑**	**—**	**↑**	**↑**	**↑↑**

*This table summarizes changes in the listed metabolic and pathologic parameters as compared to Wt mice fed chow (Wt control) in each gender.

A symbol “-”indicates no change from Wt control.

Males are more susceptible to HCFD-induced NASH due possibly to lower plasma adiponectin levels in general which may underlie their lipogenic propensity: induction of nSREBP-1c and suppression of PPARα as a consequence of reduced adiponectin signaling in the liver [34]. Indeed, the hepatic levels of p-AMPK, the effector intermediate molecule for anti-lipogenic adiponectin signaling, tended to be lower in males in our study. Another potential reason for the susceptibility in male mice is lower expression of M2 genes such as Arg-1 and Chi3l3 in the liver than females. When M1 genes (TNFα and MCP-1) are induced after HCFD feeding, this relatively repressed expression of M2 genes may aggravate pro-inflammatory response and the genesis of fatty liver as demonstrated by Odegaard, et al [35]. Certainly, inflammation in WAT is tightly associated with HCFD-induced NASH and aggravation of this pathology by hepatic IKKβ deficiency. In the latter condition, TNFα and NOS2 expression were most upregulated with heightened inflammation, which could explain suppression of adiponectin expression in WAT (Fig 5J). A question arises as to why IKKβ deficiency in hepatocytes leads to more inflammation in WAT. Is it because the livers of IKKβ deficient mice are more inflamed and this causes inflammation in WAT? Or hepatic IKKβ deficiency aggravates hepatic and global IR under HCFD feeding, and this in turn worsens WAT inflammation? Although our study did not address this question, it has obvious pathogenetic significance and warrants future investigations.

Our attempt to gain insights into the mechanisms underlying the gender difference in how hepatic IKKβ deficiency influences HCFD-induced NASH, reveals differentially expressed genes in males as opposed to females. *Sult1e1* which encodes a sulfotransferase with high affinity to estrogen, is such example and this gene is conspicuously induced in male but not female mice due to hepatic IKKβ deficiency to the level which is ~150 fold higher than that seen in male Wt mice fed chow. This also translated to the highest activity of this enzyme in HCFD-fed male mice with hepatic IKKβ deficiency. This induction is due partly to HCFD feeding as Wt mice fed HCFD have induced *Sult1e1* mRNA and enzyme activity in both genders (Fig 6A and 6D). Upregulation of *Sult1e1* which is responsible for sulfation and inactivation of estrogen, is reported in *db/db* mice [36], and this may be mediated by LXR-dependent transactivation [23] as LXR is frequently induced in obesity and fatty livers [4]. Indeed, we observed increased enrichment of LXRα to the *Sult1e1* promoter in the livers of HCFD-fed male Wt and IKKβ deficient mice (Fig 6E). We also show that suppression of NF-κB promotes LXRα-dependent *Sult1e1* promoter activity, and this most likely contributes to highly upregulated *Sult1e1* and its enzymatic activities in HCFD-fed male IKKβ deficient mice. There appears to be reciprocal antagonism between NF-κB and LXR. LXR agonists inhibit many NF-κB responsive genes via a mechanism which appear to acts downstream of NF-κB binding to DNA [37]. Conversely, agonists such as LPS and cytokines which activate NF-κB are known to repress LXR expression [38]. Thus, the deficiency of IKKβ in hepatocytes may de-repress LXR expression resulting in enhancement of HCFD-induced *Sult1e1* expression. This in turn, lowers circulating levels of estradiol and compromises hepatoprotective effects of this hormone in HCFD-fed male mice deficient in IKKβ in hepatocytes. Why this effect is not observed in female mice is an important question for which we cannot offer any concrete notions presently, but it may be related to the fact that *Sult1e1* is expressed and regulated in a sexually dimorphic manner [39].

In summary, our results revealed that the gender profoundly influences the way IKKβ in myeloid cells or hepatocytes regulates the genesis or severity of HCFD-induced obesity, IR, and NASH. Although myeloid IKKβ deficiency was beneficial for the most if not all of the metabolic consequences of HCFD feeding in both genders, hepatocyte IKKβ deficiency was detrimental in male but not female mice. This finding underscores the importance of careful consideration for pharmacological IKKβ inhibition advocated for the treatment of NAFLD and NASH [40] as this systemic approach most likely results in harmful effects of hepatocyte IKKβ inhibition counteracting beneficial effects on myeloid cells.

## Supporting information

S1 Table Genes up-regulated by hepatocyte IKKβ deficiency in males but not in females. (XLS)

S2 Table Genes down-regulated by hepatocyte IKKβ deficiency in males but not females. (XLS)
